# Coping with Stress, Executive Functions, and Depressive Symptoms: Focusing on Flexible Responses to Stress

**DOI:** 10.3390/jcm10143122

**Published:** 2021-07-15

**Authors:** Tsukasa Kato

**Affiliations:** Department of Social Psychology, Toyo University, 5-28-20 Hakusan, Bunkyo-Ku, Tokyo 112-8606, Japan; mtsukasa@hotmail.com; Tel.: +81-3-3945-4542; Fax: +81-3-3945-7626

**Keywords:** coping flexibility, inhibition, set-shifting, dual-process theory, depression, executive function

## Abstract

Coping flexibility is conceptually similar to both inhibition and set-shifting. Though they serve different functions, all three are robustly associated with depression. Coping flexibility is the ability to relinquish a coping strategy regarded as ineffective and to devise and implement an alternative one; the concept is based on stress and coping theory. Inhibition is the ability to suppress responses selectively according to a change in the situation, while set-shifting is the process of switching flexibly between task sets, mental sets, or response rules. Inhibition and set-shifting are both executive functions in cognitive mechanisms. We hypothesized that coping flexibility was associated with a lower risk of depression, even when the effects of inhibition and set-shifting were controlled for. In total, 200 Japanese university students (100 women and 100 men) completed questionnaires that measured coping flexibility and depression and performed the Stroop Color and Word Test and the Wisconsin Card Sorting Test, which measured inhibition and set-shifting. We found that greater coping flexibility was associated with a lower risk of depression, even when the effects of inhibition and set-shifting were controlled for. Our findings suggest that, although coping flexibility is conceptually similar to inhibition and set-shifting, its association with depression differs from theirs.

## 1. Introduction

Depression is a leading cause of both disease burden and years of life lived with disability worldwide. Unipolar depressive disorders account for 8.6% of total disability-adjusted life years [1]. Furthermore, the prevalence of depression is higher than that of other disabilities; the World Health Organization’s World Mental Health surveys found a 12-month prevalence of 2.2% in a Japanese community sample [2].

The accumulated evidence suggests that the onset and recurrence of depression are preceded by chronic stressors, which individuals experience repeatedly in daily life [3,4,5,6]. The vulnerability-stress (or diathesis-stress) model of depression, one of several stress models that associates chronic stress with the onset and recurrence of depression, hypothesize that individual differences in responses to chronic stressors are associated with differing vulnerabilities to depression; this hypothesis is supported by a large body of numerous neurobiological, epigenetic, and psychosocial research [7,8,9]. This study examined how coping flexibility and executive functions, which are conceptually similar in terms of vulnerability to depression, differ in their relationships to depression.

### 1.1. Coping Flexibility and Depression

The dual-process theory of coping flexibility [10,11] defines it as the ability to discontinue a coping strategy (which is a cognitive and behavioral effort to manage external and/or internal demands) regarded as ineffective and to devise and implement an alternative one to manage a stressor. By contrast, coping inflexibility refers to the perseveration of a failed coping strategy. Central to the concept of coping flexibility in the dual-process theory is the substitutability of a failed coping strategy. Some individuals attempt to deal with stressor using one coping strategy or more. Individuals with greater coping flexibility will relinquish a coping strategy deemed to be ineffective; this process is referred to as “abandonment.” Relinquishing an ineffective strategy prevents individuals from experiencing repeated failures, which can lead to negative emotions and harm their health [12,13]. An individual with greater coping flexibility will devise and implement alternative coping strategies after having relinquished the failed one. An alternative strategy is selected as the most appropriate from among the available options; this process is referred to as “re-coping.” Inflexibility in the selection of available strategies and the implementation of the selected one leads to psychopathology [13]. The dual-process theory is closely related to the transactional mode [14], which is a typical model in stress and coping from which the notion of coping flexibility is derived, and abandonment and re-coping processes in the dual-process theory are regarded as core components of flexibility by some researchers [13,15,16].

The coping flexibility hypothesis [10] posits that greater coping flexibility will produce more adaptive outcomes in psychological and physical responses to stressors [14,15]. This hypothesis, which is based on the principles of the dual-process theory, has been supported through studies done in multiple countries, such as the United States [17,18,19,20,21], the United Kingdom [22], Poland [23,24], Australia [19], Canada [25], China [19,26,27], Hong Kong [28,29], Japan [10,11,30,31,32,33,34,35,36], India [20,37], Malaysia [38], and Israel [39,40]. For example, greater coping flexibility was found to be associated with lower change scores from baseline to reactivity in heart rate and systolic blood pressure responses during a stressful cognitive task, but not during a non-stressful task [33], indicating that greater coping flexibility reduces cardiovascular reactivity to a stressful task. Moreover, an intervention to obtain greater coping flexibility was found to attenuate depression and anxiety among employees [38].

The dual-process theory can be used to predict chronic symptoms caused by chronic stress. According to the dual-process theory, repeated failures in coping (rather than a single failure) lead to a prolonged stress response; repeated failures in coping increase one’s susceptibility to stressors. Furthermore, they generate other stressful events and cause repeated experiences of stressors. Depression is a principal response to chronic stress [41,42]. Many studies have demonstrated that lower coping flexibility based on the dual-process theory is associated with lower levels of depression [10,17,19,21,23,26,27,30,31,32,33,34,35,36,37]. For example, both abandonment and re-coping were found to explain a unique amount of the variance in depressive symptoms after 14 weeks from baseline, beyond coping flexibility as measured by other approaches (i.e., coping repertoire, coping variability, and coping fitness) as well as typical coping strategies [11].

### 1.2. Executive Functions and Depression

Chronic stress is known to generally impair executive functioning [43,44], the cognitive process that underlies goal-directed behavior in a flexible manner. Deficits in executive functions, particularly inhibition and set-shifting, can make it difficult for people to respond flexibly to negative stimuli or events [45,46], which is a characteristic of major depressive disorder (MDD), and this condition can persist even after the remission of depression [47]. Additionally, a cognitive model of depression [7] hypothesizes that deficits in executive functions, particularly inhibition and set-shifting, are a manifestation of cognitive distortions, which lead to depression.

Inhibition refers to the ability to suppress responses selectively according to a change in a situation; it allows one to respond flexibly to stressors by adjusting one’s behavior and emotions, such as selectively reducing negative information [48]. Deficits in inhibition are frequently observed in MDD patients and remitted MDD patients [47,49]. One meta-analytic study [45] revealed poorer performance on the Stroop Color and Word Test (SCWT)—a neuropsychological task that measures inhibition—in MDD patients relative to healthy controls (*d* = 0.74, 95% CIs [0.52, 0.96], *k* = 7, *N* = 335). Additionally, MDD patients displayed a weaker inhibition response to negative information, but not to neutral or positive information, than nondepressed anxious patients and healthy controls [50].

Set-shifting involves the ability to switch flexibly between task sets, mental sets, or response rules. Deficits in set-shifting have been repeatedly found in MDD patients and remitted MDD patients [45,46,47], particularly in neuropsychological studies. In a meta-analytic study [51], first-episode MDD patients exhibited lower performance in set-shifting tests (Hedges’ *g* = 0.53, *k* = 7) including the Wisconsin Card Sorting Test (WCST), a neuropsychological task used to measure set-shifting. For example [52], MDD patients exhibited a higher number of perseverative errors for negative words, but not for positive or neutral words, in a WCST-type task than did healthy controls.

### 1.3. Coping Flexibility and Executive Functions

Inhibition and set-shifting are conceptually similar to coping flexibility in the dual-process theory in several ways. Both concepts focus on the relinquishment of a failed or undesirable strategy, as coping flexibility does. However, the concept of set-shifting includes the selection of an alternative strategy. Inhibition is conceptually similar to abandonment and set-shifting is similar to the concepts of united abandonment and re-coping. In fact, studies [10,11] have shown that greater cognitive flexibility, as measured by a self-report questionnaire, was associated with higher abandonment (small-to-medium effect sizes) and re-coping (medium-to-large effect sizes). Another study [10] demonstrated that individuals who could solve a puzzle involving an insight problem that required them to break a constrained solution space by relaxing its constraints (i.e., by shifting their thinking) reported higher scores on measures of abandonment coping and adaptive coping than did individuals who could not solve it (*d* = 0.57, *p* < 0.001, *n* = 77). This finding suggests that abandonment and adaptive coping are both strongly related to the ability to shift one’s thinking.

Although coping flexibility based on the dual-process theory is similar to inhibition and set-shifting in some respects, it differs from them theoretically. Inhibition and set-shifting studies based on neuroscience have focused on executive functions and cognitive models of depression, such as Beck’s [7]. Deficits of inhibition or set-shifting have been observed in patients with depression as an impairment of cognitive functions. However, the concept of coping flexibility is based on the transactional model [14], which is a major theory regarding stress and coping; it posits that repeated failures to cope with stressors lead to or exacerbate depression. Therefore, coping flexibility may contribute to a prediction of depression onset or recurrence, unlike inhibition and set-shifting.

We hypothesized that a higher level of coping flexibility would be associated with a low risk for depression, even when the effects of inhibition and set-shifting performance—which are related to the core concept of coping flexibility—are controlled for.

## 2. Materials and Methods

### 2.1. Participants and Procedure

The participants comprised 200 Japanese college students (100 women and 100 men) ranging in age from 18 to 25 years (mean 20.05 years, SD = 0.93), excluding 6 potential participants who dropped out. After signing an informed consent form, the participants completed the questionnaires designed to measure coping flexibility, depression, and several lifestyle-related questions. They entered our laboratory and were then individually administered the SCWT and WCST using a computer. The participants received no compensation for their participation.

### 2.2. Materials

#### 2.2.1. Coping Flexibility

Coping flexibility was measured with the Japanese versions of the abandonment and re-coping subscales of the Coping Flexibility Scale-Revised [11], which was designed to measure coping flexibility based on the dual-process theory. Sample items for the abandonment and re-coping subscales (five item pers subscale) included “I do not repeat using a coping strategy that made the situation worse” and “Even if the stressful situation has worsened, I can cope by using another strategy,” respectively. Both subscales of the Japanese version were correlated with higher scores on several flexibility and conceptually related variables that predicted lower levels of depression and general distress [11]. The Cronbach’s alphas found in a meta-analysis (*k* = 9, *N* = 6,752) using the college student sample were 0.87 (95% CIs [0.87, 0.88]) for abandonment and 0.92 (95% CIs [0.91, 0.92]) for re-coping [11]. The participants were required to rate the extent to which each item applied to them on a 4-point Likert-type scale ranging from 0 (*not applicable*) to 3 (*very applicable*). Higher scores indicated greater coping flexibility. In our study, the Cronbach’s alphas were 0.73 for abandonment and 0.87 for re-coping.

#### 2.2.2. Depression

The Japanese version [30] of the Center for Epidemiological Studies Depression Scale (CES-D) [53] was used to measure depressive symptoms. The Cronbach’s alpha for the Japanese version of the scale was 0.92 in the college student sample [30]. The participants rated each item according to their experiences within the past week on a 4-point Likert-type scale ranging from 0 (*rarely or none of the time*; *less than 1 day*) to 4 (*most or all of the time*; *5–7 days*). The Cronbach’s alpha in our study was 0.79.

This study used a CES-D cut-off score of 27 to identify the portion of the sample with depression. Although a CES-D score of 16 has been used as the traditional cut-off point for detecting depression, the CES-D scores reported in Japanese college students are often higher. For example, Japanese college students (*N* = 1770) reported that the prevalence of depression using a cut-off point of 16 was 56.95% (95% CIs [54.58, 59.27]), with a mean CES-D score of 19.77 (SD = 16.67) [19]. Therefore, other cut-off points have also been used. For example, some studies selected a cut-off point of 27 as an indicator of moderate-to-severe depression [19,54]; using a CES-D score of 27 identified a depression prevalence among Japanese college students of 28.53% (95% CIs [26.55, 30.62]) [19].

#### 2.2.3. Inhibition

Inhibition was assessed with the SCWT. Golden’s [55] standard stimulus material (red, green, and blue) and timing (45 s) were employed to conduct the test, which involved the presentation of three cards: a word (W) card, a color (C) card, and a color-word (CW) card. In this study, a female experimenter instructed participants to read words or identify colors as rapidly and accurately as possible, and another female experimenter recorded the participants’ responses. The Stroop interference score (I_G_) was calculated using Golden’s method: I_G_ = CW score minus P_CW_, P_CW_ = 45/{[(45 × W score) + (45 × C score)]/(W score × C score)}. Golden’s method has been used widely and frequently as an indicator of inhibition performance [56]. A lower I_G_ indicates greater difficulty in inhibiting interference.

#### 2.2.4. Set-Shifting

Set-shifting was assessed using the WCST. The computer version of WCST 4 [57] requires participants to sort one of 128 response cards with one of a set of 4 stimulus cards that differ in color, the shape depicted, and number. Each response card can be matched to a stimulus card according to a sorting rule that switches unpredictably throughout the task. Participants must infer the sorting rule through feedback regarding whether the selected stimulus card is correct. This study focused on the number of perseverative errors (WCST PE)—errors wherein a participant used the same rule for his or her choice as was used for the previous choice, but also presented non-perseverative errors (WCST NPE), which are all the remaining incorrect responses other than the perseverative errors above—as references for future research. Higher scores on the WCST PE indicate poor set-shifting performance.

### 2.3. Data Analysis

A logistic regression analysis was conducted to compute the adjusted odds ratios (ORs) associated with depression. The prevalence of depression was determined using 95% confidence intervals (CIs). Gender, SCWT I_G_ score, WCST PE and NPE scores, and abandonment and re-coping scores were entered into the regression equation. Statistical analyses were performed using SPSS version 22 (IBM Corporation, Armonk, NY, USA).

## 3. Results

The proportion of women (17.00%, 95% CIs [10.10, 24.73]) with depression (identified using a CES-D score of 27 as the cut-off point) was higher than that of men (12.00%, 95% CIs [5.77, 18.92]). The means of all variables and the differences in each variable between participants with depression and those without (i.e., individuals without depression minus those with depression) are shown in Table 1. The means of abandonment, re-coping, and SCWT I_G_ scores among the participants without depression were significantly higher than those among the participants with depression. The mean WCST PE score for the participants without depression was significantly lower than that for participants with depression. However, the difference in the WCST NPE score was non-significant. These results were consistent with our expectations.

The logistic regression analysis revealed that the model was significant, *χ*^2^ (6, *N* = 200) = 39.04, *p* < 0.001, Nagelkerke *R*^2^ = 0.32. As shown in Table 2, the coping flexibility scores of both abandonment (OR = 0.80, 95% CIs [0.64, 0.99], *p* = 0.043) and re-coping (OR = 0.77, 95% CIs [0.62, 0.95], *p* = 0.014) were significant. Significant SCWT I_G_ (OR = 0.92, 95% CIs [0.88, 0.97], *p* = 0.001) scores were also found. However, the WCST PE and WCST NPE scores were not significant.

Finally, the SCWT scores were positively and significantly correlated with the abandonment scores (*r* = 0.21, *p* = 0.003). The WCST PE scores were negatively and significantly correlated with the abandonment scores (*r* = − 0.17, *p* = 0.016), whereas they were negatively and non-significantly correlated with the re-coping scores (*r* = 0.06, *p* = 0.437)(see Table S1 in the Supplementary Material).

## 4. Discussion

As predicted, the two processes of coping flexibility—abandonment and re-coping—were both found to be associated with a lower risk for depression. Additionally, each effect size was moderate, particularly the effect size for re-coping approximated to large. Moreover, the logistic regression analysis showed that greater abandonment and re-coping were associated with a lower risk of depression, even when the effects of inhibition and set-shifting—operatively defined by the SCWT I_G_ and the WCST PE, respectively—were controlled for. Our hypothesis was thus supported in our sample.

The dual-process theory predicts that coping inflexibility will increase the risk of depression, as the persistence in a failed coping strategy prolongs the stress response and leads to chronic stress; depression will ultimately appear as a chronic stress response. Therefore, depression may be attenuated by improving coping inflexibility. However, this study was unable to examine the causal direction between coping flexibility and depression because it used a cross-sectional design. The opposite causal direction is also plausible: Coping flexibility may decrease as a result of serious depression or some individuals with depression may have coping inflexibility as a depressive symptom. These are not groundless conjectures. Some researchers, particularly in the neuroscience field, consider a deficit of inhibition or set-shifting as a symptom of depression [45,47]. In either case, even if coping inflexibility is improved, depression in unlikely to be attenuated because improving coping inflexibility does not eliminate the cause of a depression.

We consider that the causal direction of the former is valid, while that of the latter is not. Several studies [58] have demonstrated that depression can be reduce by improving coping inflexibility. For example, one study [18] found that coping flexibility, measured based on the dual-process theory, increased through mediation training. Another study [38] in which the effect size was large found that an intervention aimed at providing employees with greater coping flexibility, as defined by the dual-process theory, attenuated their depression—a result that decreased employee absenteeism, and increased presenteeism.

We argued that inhibition is conceptually similar to abandonment, and that set-shifting is similar to abandonment and re-coping. However, our study showed that, although abandonment was correlated with inhibition and set-shifting, the effect sizes were small. Additionally, re-coping was not significantly correlated with set-shifting, as measured by the WCST. The WCST is a cognitive task that measures the ability to switch between task sets, but not under stress, whereas abandonment and re-coping involve the ability to change strategies as a response to stress. Switching between strategies under stress may be a different function from that performed under nonstress conditions; the same is true for the SCWT. Moreover, the WCST may not be a pure test of set-shifting. The WCST has long been considered a distinctive test of prefrontal cortex function, which is strongly related to set-shifting. However, this idea has recently attracted much criticism [59]. Recent clinical research has concluded that poor WCST performance does not occur exclusively under prefrontal cortex deficit conditions and that the WCST is not a pure test of set-shifting [60].

Although abandonment and re-coping are conceptually similar to inhibition and set-shifting, coping flexibility serves a function that differs from those served by inhibition and set-shifting. Additionally, coping flexibility is a unique concept that is different from the concepts of inhibition and set-shifting. Although the concept of inhibition includes the suppression of a failed or a maladaptive response, it is unsuitable as an indicator of the perseveration of a failed strategy. For example, the SCWT I_G_ used in this study does not measure the perseveration of a failed strategy. The SCWT I_G_ is an indicator that inhibits the cognitive interference of words or colors, and it is not directly related to the perseveration of a failed strategy. Inhibition as measured in this study is very different from abandonment. From this perspective, abandonment is conceptually close to the WCST PE, an error in which a participant continues to follow the same rule as the SCWT I_G_.

Set-shifting may be involved in both the discontinuation of a failed strategy and the execution of an alternative strategy; however, these is no differentiation between the two processes. In other words, in set-shifting, the discontinuation of a failed strategy and the execution of an alternative strategy are not treated as different concept. As a result, the two are measured as one score, such as the WCST PE. By contrast, coping flexibility measured on the basis of the dual-process theory can be estimated separately. This characteristic of coping flexibility may contribute to the development of interventions for attenuating depression. For example, our logistic regression showed that, although the effect of set-shifting performance on the risk of depression disappeared when the effect of inhibition was controlled for, the effect of abandonment (or re-coping) was associated with a risk of depression even when the effect of re-coping (or abandonment) was controlled for. This finding may suggest the importance of understanding the two concepts (including through measurements) by distinguishing and defining them more clearly.

Finally, interventions aimed at enhancing coping flexibility via psychotherapy (rather than pharmacotherapy) may be an appropriate treatment for depression. In fact, some studies [38] found that an intervention for improvement of coping flexibility based on the dual-process theory reduced depressive symptoms. Additionally, another study [61] found such intervention could help the clinical population, such as anorexia nervosa. Our findings may thus advance the understanding of the mechanism by which psychotherapy can lead to biological changes in the central nervous system, which is a major object of this Special Issue of *The Journal of Clinical Medicine*.

### Limitations

This study has several limitations. First, we assessed inhibition and set-shifting using SCWT and WCST, respectively, which are commonly used to assess inhibition and set-shifting in neuroscience research on depression. However, some studies have assessed inhibition and set-shifting using multiple tasks. This method might have provided a better measurement of inhibition and set-shifting, as we used only one task each to minimize participant burden (Each task (i.e., the SCWT and WCST) took approximately 20 to 40 min to complete).

Second, our sample was limited to a non-clinical population (i.e, Japanese college students) who were not patients with depression. Additionally, our sample was screened for depression using a self-report scale without being really diagnosed. However, the coping flexibility hypothesis based on the dual-process theory has been supported by studies that have been conducted in various countries and that have examined various types of samples, such as individuals with chronic pain [23,31,32,36], schizophrenia [40], and anorexia nervosa [61]. Therefore, we expect that our findings will be replicated in future research using different samples.

Finally, as discussed above, our study used a cross-sectional design; therefore, the causal direction of the association between coping flexibility and depression—as well as that between inhibition and set-shifting and depression—could not be examined.

## 5. Conclusions

We found that a high level of coping flexibility was associated with a lower risk of depression, even when the effects of inhibition and set-shifting were controlled for. Although coping flexibility is conceptually similar to inhibition and set-shifting, its association with depression differs from those of inhibition and set-shifting. Our findings may further the understanding of the mechanism by which psychotherapy leads to biological changes in the central nervous system.

## Figures and Tables

**Table 1:** Means of all variables in each variable between participants with depression (*n* = 171) and those with non-depression (*n* = 29).

	Depression		Non-Depression				
Valiable	*M*	*SD*	*M*	*SD*	*t* Value	*p* Value	*d* Value
Abandonment	5.15	2.78	3.17	2.15	3.64	<0.001	0.73
Re-coping	6.23	2.69	4.14	2.28	3.96	<0.001	0.80
STWT I_G_	28.00	9.12	21.20	8.70	3.74	<0.001	0.75
WCST PE	9.14	12.87	16.14	19.71	2.48	0.014	0.50
WCST NPE	13.51	4.68	13.41	5.97	0.10	0.923	0.02
Depression	16.56	5.37	32.52	5.51	14.75	<0.001	2.96

Note. *N* = 200. SCWT I_G_, WCST PE, and WCST NPE are interference score of the Stroop Color and Word Test, perseverative error of the Wisconsin Card Sorting Test, and non-perseverative error of WCST, respectively.

**Table 2:** Logistic regression analysis on depression.

					95% CI		
	B	SE	Wald	OR	LL	UL	*p* Value
Abandonment	−0.22	0.11	4.09	0.80	0.64	0.99	0.043
Re-coping	−0.27	0.11	6.00	0.77	0.62	0.95	0.014
Gender	0.63	0.46	1.87	1.88	0.76	4.67	0.172
STWT I_G_	−0.08	0.03	10.34	0.92	0.88	0.97	0.001
WCST PE	0.03	0.02	3.02	1.03	0.10	1.06	0.082
WCST NPE	−0.08	0.05	2.64	0.92	0.84	1.02	0.104
Nagelkerke *R*^2^						0.32	<0.001

Note. *N* = 200. SCWT I_G_, WCST PE, and WCST NPE are interference score of the Stroop Color and Word Test, perseverative error of the Wisconsin Card Sorting Test, and non-perseverative error of WCST, respectively. CI, LL, and UL are respectively the confidence interval, lower limit, and upper limit for each difference.

## Data Availability

This manuscript’s data will not be deposited. The data are available from the author upon reasonable request.

## Supplementary Materials

The following are available online at https://www.mdpi.com/article/10.3390/jcm10143122/s1, Table S1: Zero-Order Between the Coping Flexibility Scale-Revised Scores and Other Variable Scores.
