# Supplementary material for: Coping with Stress, Executive Functions, and Depressive Symptoms: Focusing on Flexible Responses to Stress
Source: J Clin Med. 2021 Jul 15;10(14):3122. doi: 10.3390/jcm10143122 (PMC8304560; doi:10.3390/jcm10143122)
Supplement: Supplementary file 1 [file jcm-10-03122-s001.zip › jcm-1252285-supplementary.pdf]

Supplementary Table S1

Zero-Order Between the Coping Flexibility Scale-Revised Scores and Other Variable Scores.

Variable	Abandonment		Re-coping	
	r value	*p* value	*r* value	*p* value
SCWT I_G	0.208	0.003	0.087	0.220
WCST PE	-0.169	0.016	-0.055	0.437
WCST NPE	-0.143	0.044	-0.092	0.197
Depression	-0.289	< 0.001	-0.303	< 0.001

Note. SCWT and WCST is the Stroop Color and Word Test and the Wisconsin Card Sorting Test, respectively. I_G, PE, and NPE is interference score, perseverative error, and nonperseverative error, respectively.
